# A Nutrient-Tunable Bistable Switch Controls Motility in *Salmonella enterica* Serovar Typhimurium

**DOI:** 10.1128/mBio.01611-14

**Published:** 2014-08-26

**Authors:** Santosh Koirala, Patrick Mears, Martin Sim, Ido Golding, Yann R. Chemla, Phillip D. Aldridge, Christopher V. Rao

**Affiliations:** ^a^ Department of Chemical and Biomolecular Engineering, University of Illinois at Urbana-Champaign, Urbana, Illinois, USA; ^b^ Department of Physics, University of Illinois at Urbana-Champaign, Urbana, Illinois, USA; ^c^ Center for the Physics of Living Cells, University of Illinois at Urbana-Champaign, Urbana, Illinois, USA; ^d^ Verna and Mars McLean Department of Biochemistry and Molecular Biology, Baylor College of Medicine, Houston, Texas, USA; ^e^ Centre for Bacterial Cell Biology, Newcastle University, Newcastle upon Tyne, United Kingdom; ^f^ Institute for Cell and Molecular Biosciences, Newcastle University, Newcastle upon Tyne, United Kingdom

## Abstract

Many bacteria are motile only when nutrients are scarce. In contrast, *Salmonella enterica* serovar Typhimurium is motile only when nutrients are plentiful, suggesting that this bacterium uses motility for purposes other than foraging, most likely for host colonization. In this study, we investigated how nutrients affect motility in *S. enterica* and found that they tune the fraction of motile cells. In particular, we observed coexisting populations of motile and nonmotile cells, with the distribution being determined by the concentration of nutrients in the growth medium. Interestingly, *S. enterica* responds not to a single nutrient but apparently to a complex mixture of them. Using a combination of experimentation and mathematical modeling, we investigated the mechanism governing this behavior and found that it results from two antagonizing regulatory proteins, FliZ and YdiV. We also found that a positive feedback loop involving the alternate sigma factor FliA is required, although its role appears solely to amplify FliZ expression. We further demonstrate that the response is bistable: that is, genetically identical cells can exhibit different phenotypes under identical growth conditions. Together, these results uncover a new facet of the regulation of the flagellar genes in *S. enterica* and further demonstrate how bacteria employ phenotypic diversity as a general mechanism for adapting to change in their environment.

## IMPORTANCE

Many bacteria employ flagella for motility. These bacteria are often not constitutively motile but become so only in response to specific environmental cues. The most common is nutrient starvation. Interestingly, in *Salmonella enterica* serovar Typhimurium, nutrients enhance the expression of flagella, suggesting that motility is used for purposes other than foraging. In this work, we investigated how nutrients affect motility in *S. enterica* and found that nutrients tune the fraction of motile cells within a population. Using both experimental and mathematical analysis, we determined the mechanism governing this tunable response. We further demonstrated that the response is bistable: that is, genetically identical cells can exhibit different phenotypes under identical growth conditions. These results reveal a new facet of motility in *S. enterica* and demonstrate that nutrients determine not only where these bacteria swim but also the fraction of them that do so.

## INTRODUCTION

Bacteria employ a number of different strategies for responding to changes in their environment. A prominent example is motility, which enables bacteria to move from less favorable environments to more favorable ones. This process has been studied extensively in *Escherichia coli* and *Salmonella enterica* serovar Typhimurium, two closely related bacterial species. These bacteria move by rotating left-handed helical flagellar filaments (1). Their motility systems, including the chemotaxis pathways that govern them, are nearly identical. They principally differ in how the associated genes are expressed in response to different cellular and environmental cues (2). Motility is not constitutive in these bacteria but rather is induced in response to specific signals. How these bacteria respond to these signals presumably reflects differences in how they employ motility.

Nutrients provide one example. In *E. coli*, nutrients inhibit the expression of the motility genes (3). The mechanism is governed in part by the cyclic AMP (cAMP) receptor protein (CRP) involved in carbon catabolite repression (4). The cAMP-CRP complex positively regulates the transcription of the *flhDC* operon, which contains the genes encoding the master flagellar regulator, FlhD_4_C_2_ (5). When glucose concentrations are high, *flhDC* expression is repressed, as cAMP levels are low. Conversely, when glucose concentrations are low, *flhDC* expression is enhanced, as cAMP levels are high. In *S. enterica*, nutrients enhance the expression of the motility genes (6). The mechanism involves the protein YdiV, which binds FlhD_4_C_2_ and prevents it from activating its target promoters. In addition, YdiV promotes the degradation of FlhD_4_C_2_ through the protease ClpXP (7). Nutrients repress the expression of YdiV, in part, through the action of the mRNA-binding protein CsrA, which is involved in regulating central carbon metabolism (8). When the nutrient level is high, YdiV expression is repressed, leading to enhanced expression of the motility genes. Conversely, when the nutrient level is low, YdiV expression is enhanced and expression of the motility genes is repressed. The cAMP-CRP complex also regulates the transcription of the *flhDC* operon in *S. enterica* (9), although YdiV apparently masks the effect, at least under the conditions in which these experiments were performed.

**FIG 1:**
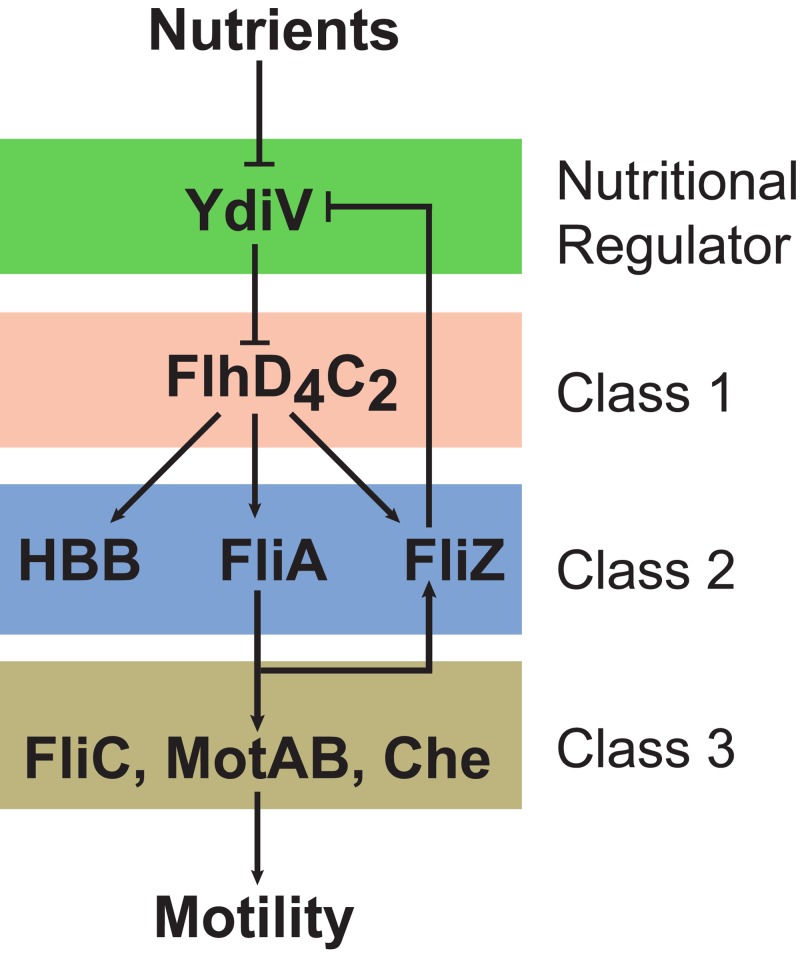
Schematic of flagellar gene network. The flagellar genes can be arranged into three classes based on how they are transcriptionally regulated (56, 57). The sole class 1 operon encodes the FlhD_4_C_2_ master regulator. FlhD_4_C_2_ activates the expression of class 2 operons, which encode the hook-basal-body (HBB) proteins. In addition, FlhD_4_C_2_ activates the expression of the alternate sigma factor, FliA (also known as σ^28^), and FliZ. FliA, in turn, activates the expression of the class 3 operons, which encode the motor proteins (MotAB), flagellar filament (FliC), and chemotaxis pathway (Che). YdiV binds FlhD_4_C_2_ and prevents it from activating class 2 promoters (6). In addition, YdiV promotes the degradation of FlhD_4_C_2_ via ClpXP (7). Both nutrients and FliZ repress the expression of YdiV (10).

YdiV participates in a double-negative feedback loop involving the flagellar regulator FliZ. FliZ directly represses *ydiV* transcription, and YdiV indirectly represses *fliZ* transcription through FlhD_4_C_2_ (Fig. 1) (10). FliZ and YdiV have also been shown to influence the population dynamics of flagellar gene expression. In particular, multiple studies have observed coexisting populations of motile and nonmotile cells (11–14); however, these coexisting populations are not observed in Δ*fliZ* (12) or Δ*ydiV* (13) mutants. Based on these results, FliZ and YdiV have been hypothesized to function in a genetic on-off switch, causing some cells to be motile and others not (15).

Although this mechanism is appealing, it has yet to be proven. Moreover, we previously found that these coexisting populations are transient, so that the entire population eventually becomes motile (12). However, these experiments were performed in rich media. One aspect that has yet to be explored is the role that nutrients play in shaping this dynamic response.

In this study, we investigated how nutrients tune flagellar gene expression dynamics in *S. enterica*. Our results demonstrate that nutrients tune the fraction of motile cells. While coexisting populations are observed at all nutrient concentrations, they persist only at intermediate nutrient concentrations. Using a combination of experimentation and mathematical modeling, we further investigated the mechanism that governs this tunable response. In support of previous models, we found that FliZ and YdiV are necessary for the response. In addition, we found that the positive feedback loop involving FliA is required, although its role appears to be solely to enhance FliZ expression. Together, these results reveal a new facet of motility and flagellar gene regulation in *S. enterica*.

## RESULTS

### Nutrients tune the fraction of motile cells.

**FIG 2:**
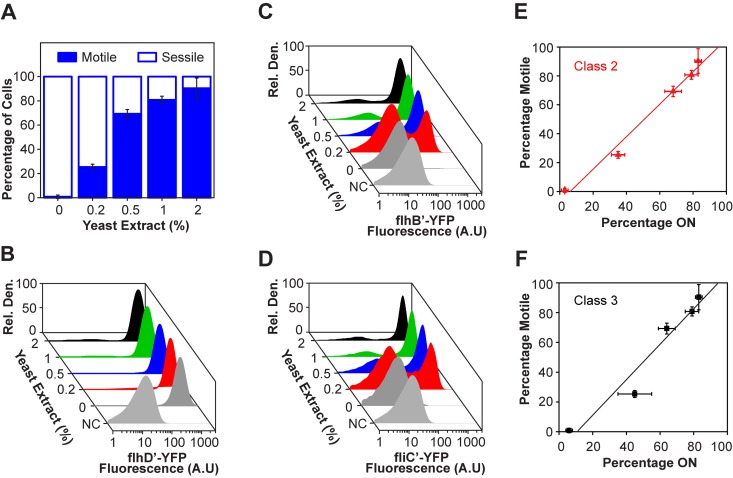
Nutrients tune the fraction of motile cells in *S. enterica*. (A) Fraction of motile cells as a function of nutrient concentrations, as determined by video microscopy. Data are averages from three independent repeats, and error bars indicate standard deviations. (B to D) Flagellar gene expression as determined using single-copy transcriptional fusions to the fluorescent protein Venus for representative class 1 (P_*flhD*_), class 2 (P_*flhB*_), and class 3 (P_*fliC*_) promoters (strains CR1404, CR1405, and CR1406, respectively). The negative controls (NC) are the Δ*flhDC* mutant (strain CR1413, Δ*flhDC*::*tetRA* uninduced). (E and F) Scattered plot of percentage of motile cells versus percentage of cells expressing class 2 and class 3 flagellar genes. Straight lines are linear fits to the data. Error bars indicate standard deviations for three independent repeats.

Previous experiments investigating the dynamics of flagellar gene expression in *S. enterica* were performed in rich Luria-Bertani (LB) medium. Based on the recent discovery that nutrients tune YdiV expression (6), we hypothesized that nutrients may also tune the fraction of motile cells. To test this hypothesis, we grew cells in Vogel-Bonner medium E (16) supplemented with 0.2% (wt/wt) glucose and various concentrations of yeast extract. The cells were harvested during late exponential phase, and their swimming behavior was analyzed by video microscopy. Consistent with our hypothesis, we found that nutrients, specifically yeast extract, tune the fraction of motile cells (Fig. 2A). We also performed growth experiments. Except in its complete absence, we found that the concentration of yeast extract does not strongly affect the growth rate (see Fig. S1 in the supplemental material). These results show that the response to yeast extract at the concentrations tested is not determined by the growth rate but is instead regulated by nutrient availability.

### Nutrients tune the fraction of cells expressing flagellar genes.

We next investigated whether regulation occurs at the level of gene expression. Flagellar genes can be divided into a transcriptional hierarchy comprising three classes (Fig. 1). We used flow cytometry to measure the expression from a representative promoter from each hierarchical class, using single-copy, chromosomally integrated transcriptional fusions to the fluorescent protein Venus (17). The class 1 P_*flhDC*_ promoter was active in all cells irrespective of yeast extract concentrations (Fig. 2B). However, we found that the class 2 P_*flhB*_ and class 3 P_*fliC*_ promoters were active in only a subpopulation of cells at intermediate yeast extract concentrations, giving rise to a bimodal distribution (Fig. 2C and D). The active fraction increased with yeast extract concentrations in a manner consistent with the video microscopy experiments (Fig. 2E and F). We note that nutrients increased the relative expression of the P_*flhB*_ and P_*fliC*_ promoters in cells where the promoters were active, though the effect is minor. As the P_*flhB*_ and P_*fliC*_ promoters are nearly identical in their response to yeast extract, our remaining investigations focused on the class 2 P_*flhB*_ promoter.

**FIG 3:**
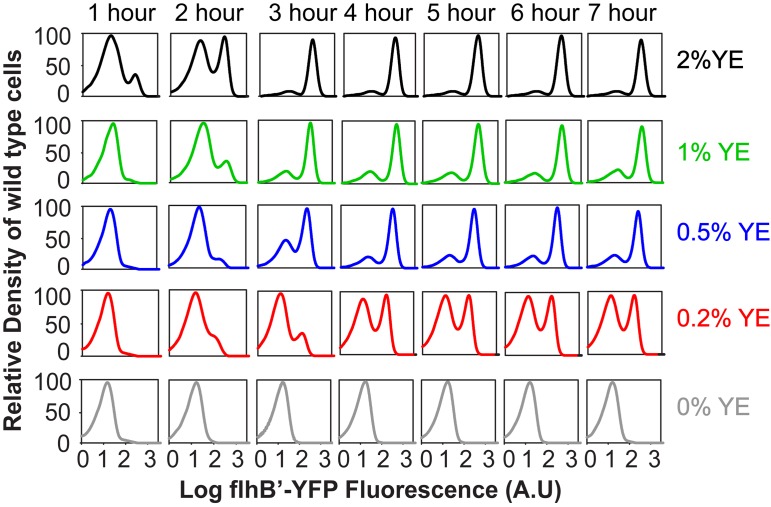
Dynamic activity of a representative class 2 (P_*flhB*_) promoter, presented as a function of time and yeast extract concentration, as determined by flow cytometry in wild-type cells (strain CR1405).

In dynamic gene expression experiments, coexisting populations of cells with active and inactive promoters were transiently observed at all yeast extract concentrations (Fig. 3). However, the population with inactive promoters persisted only at low yeast extract concentrations. At the higher concentrations, the promoters in all cells eventually became active. These results are consistent with our previous findings, in which we observed transient heterogeneity in nutrient-rich media (12). At the lower concentration of yeast extract, the coexisting populations persist for many hours with no significant change in their distribution. These results suggest that the observed response is bistable: that is, genetically identical cells can exhibit different phenotypes that persist under identical growth conditions.

### Multiple nutrients activate flagellar gene expression.

Yeast extract is a complex mixture containing many nutrients. We tested a number of different compounds for their ability to activate the P_*flhB*_ promoter (Table 1). Among the compounds tested, we found that amino acids were able to activate the P_*flhB*_ promoter, although not to the same degree that yeast extract was able to. Analysis of individual amino acids suggested that most were able to activate the P_*flhB*_ promoter weakly, whereas a combination of all twenty was able to activate the P_*flhB*_ promoter to roughly half the level achieved with yeast extract. These results indicate that the activating signal is not a simple compound but rather a mixture of them, of which amino acids are a subset. We also note that previous studies have shown that that the RNA-binding protein CsrA, which is involved in carbon storage, regulates YdiV translation, though YdiV is still subject to nutritional regulation in a Δ*csrA* mutant (6). CsrA is regulated by CsrB and CsrC, two noncoding RNAs that are transcriptionally regulated by the BarA/SirA two-component signal transduction system (18, 19). One study found that formate and acetate regulate CsrB transcription through BarA and SirA, respectively (20). We also tested the ability of formate and acetate to activate the P_*flhB*_ promoter and found that neither was able to do so (Table 1). As we were unable to isolate a single activating compound, we employed yeast extract in the remainder of our studies.

**TABLE 1:** Normalized P_*flhB*_ promoter activity with different supplements to MG media

Supplement	Concn (mM)	Fluorescence (AU)^*a*^
2% yeast extract		100
All amino acids^*b*^		53
No amino acid		13
Glycine	10	22
Alanine	10	15
Serine	10	22
Threonine	10	29
Cysteine	0.8	28
Valine	10	13
Leucine	10	18
Isoleucine	10	22
Methionine	10	28
Proline	10	30
Phenylalanine	10	16
Tyrosine	2	28
Tryptophan	10	20
Aspartic acid	10	17
Glutamic acid	10	18
Asparagine	10	17
Glutamine	10	24
Histidine	10	20
Lysine	10	20
Arginine	10	16
Succinate	25	24
Citrate	25	24
Lactate	10	16
Formate	10	12
Acetate	10	10
Propionic acid	10	22
Butyric acid	10	25
Indole	1	20

^a^ AU, arbitrary units.

^b^ All amino acids at the concentrations listed.

### YdiV and FliZ are necessary for bimodal flagellar gene expression.

**FIG 4:**
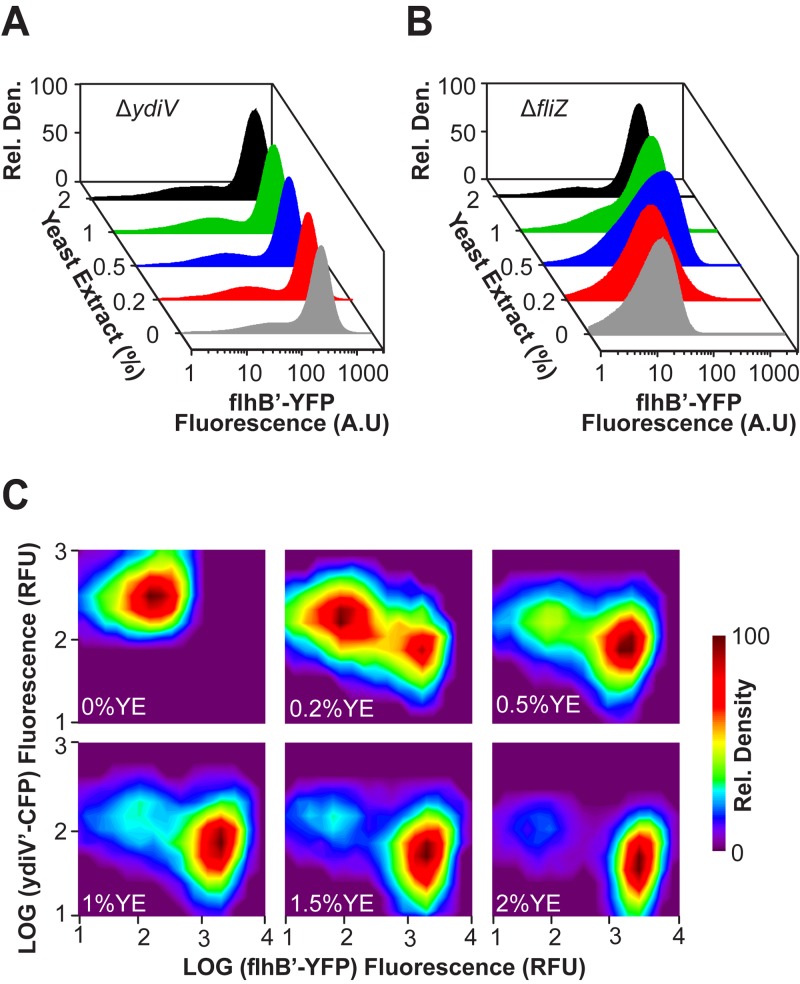
FliZ and YdiV are necessary for bimodal gene expression. (A and B) Class 2 gene expression as determined using single-copy transcriptional fusions to the fluorescent protein Venus in Δ*ydiV* (A) and Δ*fliZ* (B) mutants (strains CR1407 and CR1408, respectively). (C) Simultaneous measurement of representative class 2 (P_*flhB*_) and YdiV promoters, as determined using two-color flow cytometry (P_*ydiV*_-CFP plasmid in strain CR1405).

Both YdiV and FliZ have previously been shown to affect single-cell gene expression dynamics (12, 13). To test how these two proteins contribute to the nutritional response to yeast extract, we measured flagellar gene expression in Δ*ydiV* and Δ*fliZ* mutants. In a Δ*ydiV* mutant, the P_*flhB*_ promoter was strongly active in most cells, irrespective of yeast exact concentration (Fig. 4A; see also Fig. S2A in the supplemental material). In fact, yeast extract had no substantive effect on P_*flhB*_ promoter activity. We note that there is a tail in the distribution, indicating that the P_*flhB*_ promoter is weakly active in a small population of cells. This population was observed at all yeast extract concentrations and was also present in the wild type, even at the highest concentration of yeast extract employed (Fig. 2C and D). In contrast, the Δ*fliZ* mutant exhibited a homogeneous nutrient response consisting of a single population (Fig. 4B; see also Fig. S2B in the supplemental material). We also found that *ydiV* was dominant, so that a Δ*ydiV* Δ*fliZ* double mutant was indistinguishable from the Δ*ydiV* single mutant (see Fig. S2C in the supplemental material)*.*

YdiV and FliZ are known to repress each other, with FliZ directly repressing *ydiV* transcription and YdiV indirectly repressing *fliZ* transcription via FlhD_4_C_2_ (Fig. 1) (10). To observe this competitive interaction at single-cell resolution, we employed two-color flow cytometry to measure simultaneous expression from the P_*ydiV*_ and P_*flhB*_ promoters. Expression from the P_*ydiV*_ promoter was measured using a transcriptional fusion to the cyan fluorescent protein (CFP). Note that the P_*ydiV*_-*cfp* fusion was active only when expressed from a low-copy-number (pSC101* origin of replication) plasmid; single-copy transcriptional fusions, like those employed for the P_*flhB*_ and P_*fliC*_ promoters, were not sufficiently active to measure a response for the P_*ydiV*_ promoter.

Figure 4C shows a comparison of P_*flhB*_ and P_*ydiV*_ promoter activities as a function of yeast extract concentrations. In the absence of yeast extract (0%), a single population was observed in which the P_*ydiV*_ promoter is active and the P_*flhB*_ promoter is inactive. At intermediate concentrations of yeast extract (0.2 to 1%), two populations were observed: one in which the P_*ydiV*_ promoter was active and the P_*flhB*_ promoter was inactive and one in which the reciprocal pattern occurred. As yeast extract concentrations were increased, the relative number of cells occupying the population where P_*flhB*_ promoter was active increased. At a high yeast extract concentration (>1%), only the P_*flhB*_ active population was observed, although transcription from the P_*ydiV*_ promoter was still detectable. These results suggest that the heterogeneous nutrient response arises from the mutual repression of YdiV and FliZ.

We note that a previous study did not report any significant changes in *ydiV* transcription in response to nutrients. Instead, only changes in the level of YdiV protein were observed, suggesting that nutrients principally regulate YdiV via a posttranscriptional mechanism (6). We, on the other hand, found that yeast extract decreased expression from the P_*ydiV*_ promoter, indicating that the mechanism involves a significant transcriptional component. One possible explanation for this discrepancy is that the decrease in *ydiV* transcription is due to FliZ, which is more strongly expressed at high yeast extract concentrations and which is known to bind the P_*ydiV*_ promoter. We found, however, that yeast extract also decreased expression from the P_*ydiV*_ promoter in a Δ*fliZ* mutant (see Fig. S3A in the supplemental material). In both the wild type and the Δ*fliZ* mutant, we observed more than a 2-fold decrease in promoter activity at high concentrations of yeast extract. When YdiV expression was measured using a translational fusion to superfolder green fluorescent protein (SGFP), we observed similar decreases in its expression (see Fig. S3B), indicating that YdiV is regulated at the transcriptional level.

### Flagellar gene expression is bistable and exhibits hysteresis.

Our data suggest that flagellar gene expression is bistable. As bistable systems exhibit hysteresis, which reflects history dependence (21), we wished to determine how cells transition between different states of flagellar gene expression. To test whether flagellar gene expression exhibits hysteresis, we first replaced the native P_*flhDC*_ promoter with a tetracycline-inducible one, as described previously (22). We then grew cells in the presence and in the absence of 10 ng/ml anhydrotetracycline (aTc)—referred to here as the on and off states, respectively—prior to subculturing into fresh medium containing different aTc concentrations. We fixed the yeast extract concentration at 0.2%, as bistability is most pronounced at this concentration. If flagellar gene expression is bistable, then the response should be different for these two cases.

**FIG 5:**
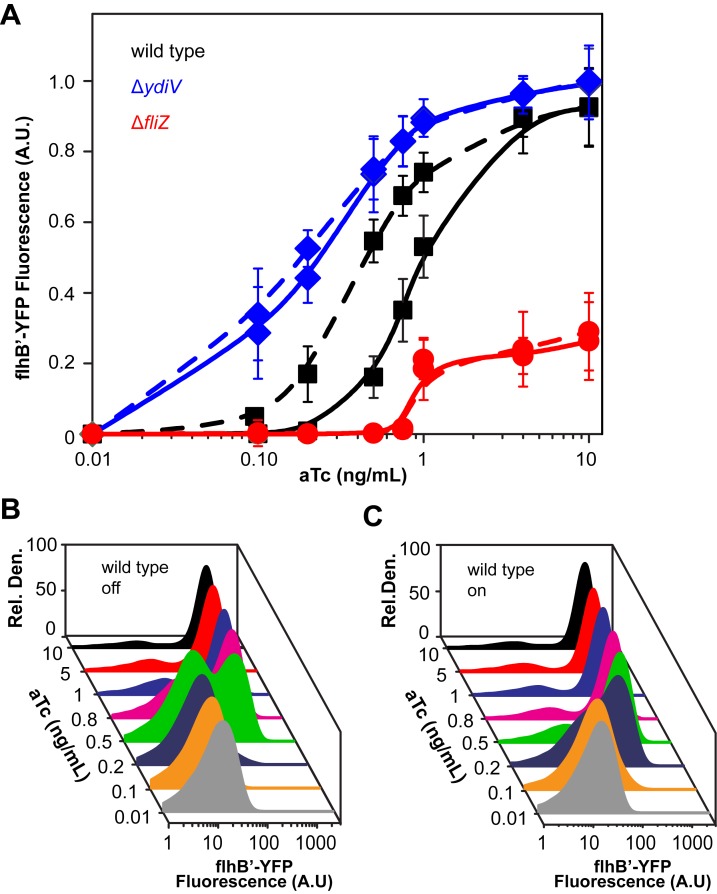
Flagellar gene expression exhibits hysteresis. (A) Experiments were performed in strains (CR1413, CR1414 [Δ*fliZ*], and CR1415 [Δ*ydiV*]) where the native P_*flhDC*_ promoter was replaced with an aTc-inducible one (P_*tetRA*_). Cells were grown in the presence (on) or absence (off) of aTc and then subcultured at intermediate aTc concentrations. Experiments were performed in 0.2% yeast extract; results for other concentrations are provided in Fig. S5 in the supplemental material. Data are averages from three independent repeats, and error bars indicate standard deviations. (B and C) Data are presented as distributions. Note that bistability is not observed during the on-to-off transition. Equivalent plots for Δ*fliZ* and Δ*ydiV* mutants are given in Fig. S4 in the supplemental material.

Consistent with a bistable response, we observed that cells exhibited different patterns of P_*flhB*_ promoter activity depending on whether they were initially in the on or off state (Fig. 5A). In general, P_*flhB*_ promoter activity was lower when cells were initially in the off state than when cells were initially in the on state. Bimodality was not observed when cells transitioned from an on to an off state (Fig. 5B and C). We performed similar experiments using the Δ*ydiV* and Δ*fliZ* mutants. For both mutants, no hysteresis was observed: the response was the same irrespective of whether they had previously been induced with aTc (Fig. 5A; see also Fig. S4 in the supplemental material). These results demonstrate that YdiV and FliZ are essential for the hysteresis response.

Similar experiments were performed at different concentrations of yeast extract, and hysteresis was again observed (see Fig. S5A to C in the supplemental material). However, we cannot directly compare these experiments with one another, as the responses to yeast extract and aTc are not orthogonal: as yeast extract concentrations increase, expression from the tetracycline-inducible P_*tetA*_ promoter (23) decreases, for unknown reasons (see Fig. S5D in the supplemental material). Despite this cross talk, the conclusions that flagellar gene expression is bistable and exhibits hysteresis do not change.

### The FliA positive feedback loop is necessary for bistability.

The flagellar network possesses two feedback loops in addition to the ones involving FliZ and YdiV. One is a negative feedback loop involving FliT. Expressed from a hybrid class 2/3 promoter (24), FliT binds to FlhD_4_C_2_ and prevents it from activating its cognate class 2 promoters (25, 26). The second is a positive feedback loop involving FliA and FlgM. The *fliAZ* operon is under the control of both class 2 and class 3 promoters (27). The class 2 promoter functions in a double-negative feedback loop involving FliZ and YdiV; the class 3 promoter functions in an autogenous loop involving the alternate sigma factor FliA. Note that the latter loop is not directly autocatalytic, as FliA is inefficiently translated from the class 3 transcript (28). Rather, positive feedback is indirect, such that FliA activates FliZ expression and FliZ indirectly activates FliA expression by repressing the expression of YdiV.

An additional facet of the regulation involves FlgM, which regulates FliA activity by binding to it and preventing it from activating its cognate class 3 promoters (29). FliA and FlgM function in a developmental checkpoint involving protein secretion (30). In addition, they are believed to function in a regulatory circuit involved in controlling the number of flagella produced per cell (31–33).

**FIG 6:**
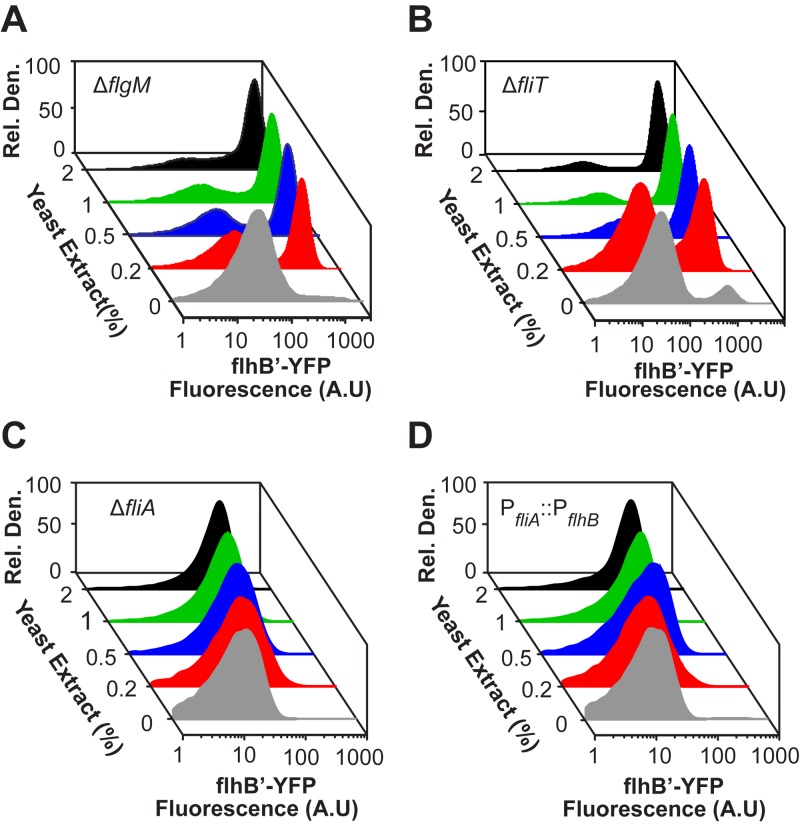
FliA positive feedback is necessary for bistability, but FlgM and FliT are not. Class 2 gene expression was determined using single-copy transcriptional fusions to the fluorescent protein Venus in Δ*flgM* (A), Δ*fliT* (B), Δ*fliA* (C), and P_*fliA*_::P_*flhB*_ (D) strains (strains CR1410, CR1411, CR1412, and CR1416, respectively).

To determine whether these regulatory loops contribute to bistability, we examined the activity of the P_*flhB*_ promoter in Δ*fliT*, Δ*fliA*, and Δ*flgM* mutants. In the case of the Δ*fliT* and Δ*flgM* mutants, the response to yeast extract was similar to that of wild-type cells (Fig. 6A and B), demonstrating that neither gene product is required for bistability. The Δ*fliA* mutant, on the other hand, exhibited a homogenous response to yeast extract (Fig. 6C) in a manner equivalent to a Δ*fliZ* mutant. We also tested a P_*fliA*_::P_*flhB*_ promoter mutant, where the hybrid class 2/3 P_*fliA*_ promoter was replaced with a pure class 2 promoter (33), and found that it also exhibited a homogeneous response to yeast extract (Fig. 6D). These results demonstrate that both FliA and the class 3 component of the P_*fliA*_ promoter are essential for bistability.

### Mathematical model reproduces bistability and provides insight regarding FliA.

We compared two simple mathematical models of the flagellar gene network to gain insight regarding the mechanism for bistability (details and equations are provided in Text S1 in the supplemental material). The first model ignores the contribution of FliA and accounts only for the interaction between YdiV and FliZ. The second model includes FliA. Both models are semiquantitative, in the sense that they were formulated solely to capture the general trends in our data rather than to reproduce them exactly.

**FIG 7:**
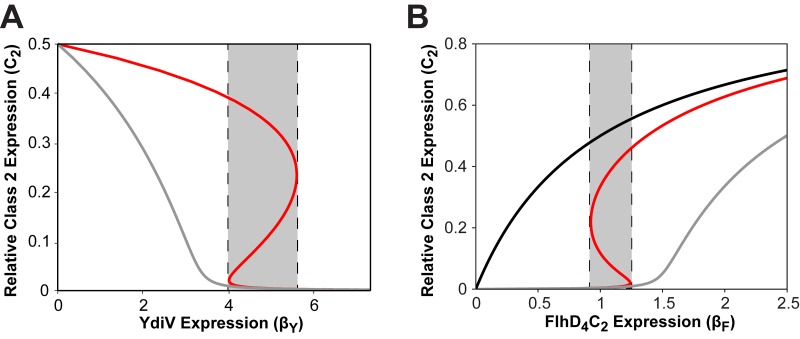
Mathematical modeling shows tfhat the interaction between FliZ and YdiV is sufficient for generating bistability. (A) The plot shows the steady-state relative class 2 gene expression (*C*_2_) as a function of the YdiV expression rate (β_Y_) for the wild type (red line). In the absence of FliZ, no bistability is observed (gray line). Note that nutrients lower the YdiV expression rate, hence the inversion of the curve relative to what is observed experimentally (Fig. 5A). (B) Model behavior as a function of the FlhD_4_C_2_ expression rate (β_*F*_) for the wild type (red line), Δ*fliZ* mutant (gray line), and Δ*ydiV* mutant (black line). The plot shows the steady-state relative class 2 gene expression (*C*_2_) as a function of the FlhD_4_C_2_ expression rate for a β_Y_ value of 5. The simulations involved the following dimensionless parameter values: β_*F*_ = 1, β_*Z*_ = 6, γ_*C*_ = 150, and γ_*C*Y_ = 500. Model results for different parameter values are provided in Fig. S6 in the supplemental material.

The first model is capable of generating bistability (Fig. 7). In our simulations, we considered two scenarios. First, we varied the level of YdiV expression in the model as a proxy for nutrient availability (Fig. 7A). For intermediate levels of YdiV expression, the model admits three steady-state solutions that represent possible states of flagellar gene expression (denoted by the shaded region in Fig. 7A). The upper and lower branches are stable and correspond to the on and off states, respectively. The middle branch is unstable and not physiological. In contrast, the model yields a single steady state when it is simulated for a Δ*fliZ* mutant (gray line in the plot in Fig. 7A) or a Δ*ydiV* mutant. In the latter, there is obviously no expression of YdiV. Simulations for different parameter values are provided in Fig. S6 in the supplemental material. Next, we performed simulations in which we varied the FlhD_4_C_2_ expression rate while keeping the YdiV expression rate fixed (Fig. 7B). These simulations were used to mimic the experiments whose results are shown in Fig. 5. The model again reproduced the general features of our experimental data, including those for the Δ*fliZ* and Δ*ydiV* mutants (Fig. 5A). These results show that positive feedback via FliA is not necessary for bistability: the regulatory interactions between FliZ and YdiV suffice.

We next considered a model that includes FliA. This second model was also capable of generating bistability (see Fig. S7 in the supplemental material). The parameters were chosen to match the response of the first model. When we compared the two models and associated parameters, the key difference was that the first model required that FlhD_4_C_2_ strongly activate the expression of FliZ to generate bistability, whereas the second model did not. Introducing FliA positive feedback relaxes the requirement for FliZ because the positive feedback functions to amplify FliZ expression by FlhD_4_C_2_ (see equation 24 in Text S1 in the supplemental material). These results are consistent with the notion that the contribution of FliA is indirect and functions to enhance FliZ expression.

## DISCUSSION

The findings presented here show that the fraction of motile cells in a population of *S. enterica* is determined, at least in part, by nutrient availability. YdiV and FliZ control two feedback loops with opposing activities that govern this response, as previously proposed (15). Although these feedback loops are necessary for bistability, they are not sufficient; a positive feedback loop involving FliA is also required. This third loop is not strictly autoregulatory, in the sense that FliA does not directly enhance its own expression.

To further understand the mechanism of bistability, we constructed a simplified mathematical model of the flagellar gene network. Analysis of this model demonstrated that, in principle, the two feedback loops controlled by FliZ and YdiV are sufficient for bistability. The positive feedback loop involving FliA, however, does not provide a critical feature for bistability and nutrient control. Rather, the FliA loop contributes to bistability by ensuring that expression of FliZ is strongly enhanced by FlhD_4_C_2_ due to *fliZ* being transcribed from a hybrid class 2/3 promoter.

These features of nutrient regulation raise the question of why *fliZ* is transcribed from a hybrid class 2/3 promoter when expression from a strong class 2 promoter would suffice to generate bistability. One potential explanation is that the FliA feedback loop couples the switch to the completion of assembly of the hook-basal body complex. A developmental checkpoint involving FliA and FlgM couples class 3 flagellar gene expression to flagellar assembly (30). Prior to completion of the hook-basal body, FlgM binds to FliA and prevents it from activating class 3 promoters. Upon completion of the hook-basal body complex, FlgM is secreted from the cell by the flagellar export apparatus, freeing FliA to transcribe from class 3 promoters. Mutations that inhibit formation of the hook-basal body complex are deficient in FlgM secretion and thus prevent transcription from class 3 promoters. This checkpoint likely ensures that cells do not switch to the on state for class 3 transcription until they are able to make functional hook-basal body complexes. In support of this prediction, we discovered that gene expression is homogenous in hook-basal body mutants (12) and in a Δ*fliA* mutant (Fig. 6C). Moreover, *fliA and fliZ* reside in the same operon in most bacteria that possess *fliZ* (34). Thus, coupling of *fliZ* and *fliA* may be necessary for the full function of FliZ to be realized.

In our work, we used yeast extract as the nutrient to tune flagellar gene expression. How yeast extract regulates the P_*ydiV*_ promoter is not known. Our work demonstrates that amino acids are able to activate the P_*flhB*_ promoter, but they are unlikely to be the only compounds that do so (Table 1). One additional possibility is that the P_*ydiV*_ promoter responds to the growth rate of the cell. Although our experiments provide no support for this possibility (see Fig. S1 in the supplemental material), a caveat is that we used relatively high concentrations of nutrients that supported approximately equal growth rates. If flagellar gene regulation also responds to the growth rate of cells, as one might expect, then our experiments would not have detected this phenomenon, as our conditions were chosen to keep the growth rate nearly constant.

The present study raises the question of why nutrients control bistable flagellar gene expression in *S. enterica*. One possible explanation derives from the fact that motility is intimately coupled to virulence and host colonization in *S. enterica*. Numerous studies have shown that flagellar gene expression is coupled to the expression of the invasion genes associated with the type III secretion system encoded within *Salmonella* pathogenicity island 1 (SPI-1) (35–40). Of note, FliZ positively regulates the expression of the SPI-1 invasion genes. In addition, flagellin activates the innate immune response (41). The nutrient response likely ensures that flagellin is expressed at specific sites within the host, as previously shown (11). Bistability, as argued by Stewart and Cookson (15), may enable a division of labor and a degree of bet hedging, where motile cells are invasive and nonmotile cells noninvasive. Nonmotile cells could thus avoid the inflammatory environment of the intestinal epithelium and serve as a reservoir for the next phases of colonization. Our results extend this model by showing that nutrients control these fractions of motile and nonmotile cells.

Nutrients repress motility in *E. coli*, a form of regulation that is consistent with *E. coli* employing motility as a foraging mechanism. Only when starved of nutrients are these bacteria motile. However, recent results suggest that motility in *E. coli* is also employed for host colonization. For example, the bacterial quorum sensing signal AI-2 and interkingdom signaling molecule norepinephrine control motility in *E. coli* (42–45). These results indicate that both *S. enterica* and *E. coli* employ motility for multiple purposes.

We note that the *ydiV* gene is also present in *E. coli* (46). Although the gene is transcriptionally active, it is poorly translated (28). However, the *E. coli ydiV* gene is efficiently translated in *S. enterica*, suggesting that some factor represses its translation in its native host. These results suggest that the flagellar gene networks in *E. coli* and *S. enterica* are quite plastic, in the sense that small changes in the expression of individual genes can result in significantly different responses to nutrients and perhaps to other environmental cues. Such plasticity in the regulatory pattern of the flagellar gene network possibly may reflect the disparate roles motility plays in these two closely related organisms and enable them to adapt readily to new environments in which these roles differ.

We conclude by noting that flagellar gene expression is also bistable in *Bacillus subtilis* (47), although the mechanism governing bistability is quite different than the one in *S. enterica* (48). In *B. subtilis*, the flagellum-specific sigma factor SigD resides at the end of a large operon containing thirty-one flagellar genes. Presumably, SigD expression is weak or nonexistent in many cells because of incomplete transcription of the full operon. In cells in which the entire operon is transcribed and expression of SigD exceeds some threshold, it can further enhance its own expression through a SigD-dependent promoter that resides in the middle of the operon. This positive feedback mechanism, which involves the stochastic triggering of the loop, generates the observed bistability in *B. subtilis* motility. Indeed, moving the *sigD* gene upstream in the operon increases the fraction of motile cells (48). Whether the bistable response in *B. subtilis* is tuned by external factors to the same degree as in *S. enterica* is not known. Nonetheless, the bistable response in these two distantly related bacteria suggests that heterogeneous expression of flagellar genes is a general phenomenon and may reflect a widespread strategy for deploying motility as an adaptational response to the environment.

## MATERIALS AND METHODS

### Media and growth conditions.

All experiments were performed at 37°C in Vogel-Bonner minimal E medium (16) supplemented with 0.2% (wt/wt) glucose and yeast extract at specified concentrations (MG medium). Luria-Bertani (LB) medium was used for strain and plasmid construction. Strains harboring plasmids pKD46, pCP20, and pINT-ts were grown at 30°C. Antibiotics were used for gene deletion and plasmid maintenance at the following concentrations: 40 µg/ml kanamycin, 20 µg/ml chloramphenicol, and 100 µg/ml ampicillin.

### Bacterial strains and plasmid construction.

All strains are derivatives of *S. enterica* serovar Typhimurium 14028 (American Type Culture Collection). The Δ*fliA* (region from 2056152 to 2056871), Δ*flgM* (region from 1215209 to 1215502), and Δ*ydiV* (region from 1432774 to 1433487) mutants were constructed using the method of Datsenko and Wanner (49). Prior to removal of the antibiotic marker, the integrated cassette was moved to a clean wild-type background by P22 transduction. The *ydiV′*-*cfp* transcriptional fusion was made by introducing the *ydiV* promoter (region from 1431646 to 1432784) and CFP from pMUTIN-CFP (50) into the plasmid pZS*31 (51) using the XhoI, EcoRI, and XbaI restriction sites. The YdiV-SGFP translational fusion was made by amplifying *ydiV* with its native promoter (region from 1431646 to 1433484) and the SGFP gene (52) with the Gly-Gly-Ser-Gly linker using overlap extension PCR (53). The PCR product was then cloned into the pVenus plasmid (54) using the SalI and NheI restriction sites and finally integrated into the chromosome using the method of Haldimann and Wanner (55). The class 1 P_*flhDC*_ promoter (region from 2034484 to 2033584), class 2 P_*flhB*_ promoter (region from 2023494 to 2022815), and class 3 P_*fliC*_ promoter (region from 2061043 to 2060527) were used as representatives for each hierarchical class of flagellar promoters. Single-copy transcriptional fusions in the chromosome were made by first cloning respective promoters into the plasmid pVenus (54) using KpnI and EcoRI restriction sites and integrating the plasmids into the chromosome using the CRIM method (55). The integrated plasmids were then moved into the wild type and the different mutants by P22 transduction. All strains and plasmids used in this work are listed in Table 2. Strain numbers are provided in the figure captions to match individual experiments to specific strain genotypes.

**TABLE 2:** Strains and plasmid used in this study

Strain or plasmid	Relevant characteristic(s)
*S. enterica* strains^*a*^	
14028	Wild type, serovar Typhimurium
CR201	Δ*fliZ*
CR1404	attλ::pVenus::P_*flhD*_-Venus
CR1405	attλ::pVenus::P_*flhB*_-Venus
CR1406	attλ::pVenus::P_*fliC*_-Venus
CR1407	Δ*ydiV* attλ::pVenus::P_*flhB*_-Venus
CR1408	Δ*fliZ* attλ::pVenus::P_*flhB*_-Venus
CR1409	Δ*fliZ* Δ*ydiV* attλ::pVenus::P_*flhB*_-Venus
CR1410	Δ*flgM* attλ::pVenus::P_*flhB*_-Venus
CR1411	Δ*fliT* attλ::pVenus::P_*flhB*_-Venus
CR1412	Δ*fliA* attλ::pVenus::P_*flhB*_-Venus
CR1413	ΔP_*flhDC*_::*tetRA* attλ::pVenus::P_*flhB*_-Venus
CR1414	ΔP_*flhDC*_::*tetRA* Δ*fliZ* attλ::pVenus::P_*flhB*_-Venus
CR1415	ΔP_*flhDC*_::*tetRA* Δ*ydiV* attλ::pVenus::P_*flhB*_-Venus
CR1416	ΔP_*fliA*_::P_*flhB*_ attλ::pVenus::P_*flhB*_-Venus
CR1417	attλ::pVenus::YdiV-SGFP
Plasmid	
P_*ydiV*_-CFP	*cm* P_*ydiV*_-*cfp* pSC101*

^a^ All strains are isogenic derivatives of *Salmonella enterica* serovar Typhimurium strain 14028 (American Type Culture Collection).

### Flow cytometry.

Cells were grown overnight at 37°C in MG medium supplemented with 0.2% yeast extract. Cells were subcultured to an optical density at 600 nm (OD_600_) of 0.05 in fresh MG medium supplemented with 0%, 0.2%, 0.5%, 1%, or 2% yeast extract. After subculture, the cells were then allowed to grow at 37°C for 4 h (unless noted otherwise) before harvesting. For dynamic gene expression experiments, samples were collected every hour for 7 h, centrifuged at 3,200 × *g* for 10 min, resuspended in phosphate-buffered saline (PBS) containing 50 µg/ml chloramphenicol, and kept on ice. The cells were pelleted by centrifugation at 3,200 × *g* for 10 min and resuspended in DAPI (4′,6-diamidino-2-phenylindole) staining buffer with 14.3 µM DAPI and 50 µg/ml chloramphenicol. The cells were then incubated at room temperature for half an hour. The cells were then analyzed using a BD LSR II flow cytometer. Fluorescence values for approximately 100,000 cells were recorded using the Pacific Blue channel (excitation, 405 nm; emission, 450/50 nm) for DAPI and the fluorescein isothiocyanate (FITC) channel (excitation, 488 nm; emission, 530/30 nm) for Venus. The cells were distinguished from other debris by gating only the population stained with DAPI. For cells expressing both CFP and Venus, the cells were not stained with DAPI; instead, the population was gated according to side scatter (SSC) and forward scatter (FSC) channels. CFP fluorescence was recorded using the Alexa Fluor 430 channel (excitation, 405 nm; emission, 525/50 nm). Data extraction and analysis for the FACS experiments were done using FCS Express version 4 (De Novo Software). The data were exported to Microsoft Excel (2010) and further processed to obtain the data for fluorescence and relative density distributions.

The hysteresis experiments were performed by first growing the cells at an initial OD_600_ of 0.05 for 3 h in 5 ml of MG medium supplemented with 0.2% of yeast extract in either the presence or absence of 10 ng/ml anhydrotetracycline (aTc). The cells were then harvested by centrifuging at 3,200 × *g* for 10 min, washed once with PBS, and finally resuspended in 50 ml MG medium supplemented with 0.2% yeast extract. Both cultures were then supplemented with various concentrations (0.01, 0.1, 0.2, 0.5, 0.8, 1, 5, and 10 ng/ml) of aTc and grown for 4 h before harvesting. The samples were stained with DAPI and analyzed with a flow cytometer as described above.

### Cell tracking.

Cells were grown overnight at 37°C in MG medium supplemented with 0.2% final concentration of yeast extract. Cells were subcultured to an OD_600_ of 0.05 in fresh MG medium supplemented with 0%, 0.2%, 0.5%, 1%, and 2% yeast extract. After subculture, the cells were then allowed to grow at 37°C for 5 h before harvesting. Glass slides and coverslips were soaked in 1 M KOH for 15 min and washed with deionized water prior to use. A 5-µl volume of appropriately diluted sample, such that there would be roughly 50 cells in the view frame, was put on a glass slide, covered, and sealed with epoxy. Cells were tracked by phase-contrast using a Zeiss standard RA microscope equipped with a Hyper HAD black-and-white video camera. The movie was then analyzed using custom MatLab software. The algorithm ignores all the cells that are stuck on the glass slides and analyzes only the cells that are swimming (motile) or drifting in the liquid (sessile).

## SUPPLEMENTAL MATERIAL

Text S1 Detailed description of mathematical models discussed in the text. Download Text S1, PDF file, 0.1 MB

Figure S1 Growth curves of wild-type cells (strain 14028) at different concentrations of yeast extract. Error bars indicate standard deviations for three independent repeats. Download Figure S1, TIF file, 0.8 MB

Figure S2 The class 2 gene expression profile is unimodal in the absence of two antagonizing proteins, YdiV and FliZ. (A to C) Class 2 P_*flhB*_ promoter activity as a function of time and yeast extract concentration, as determined by flow cytometry in Δ*ydiV*, Δ*fliZ*, and Δ*ydiV* Δ*fliZ* mutants (strains CR1407, CR1408, and CR1409, respectively). Download Figure S2, TIF file, 3.5 MB

Figure S3 (A) YdiV transcription is enhanced under nutrient-limited conditions, and its transcription is repressed by FliZ. P_*ydiV*_ promoter activity as a function of yeast extract in wild-type and Δ*fliZ* strains (P_*ydiV*_-CFP plasmid in strains 14028 and CR201, respectively). (B) YdiV is regulated at the transcriptional level. Comparison of YdiV transcriptional (ydiV*′*-CFP) and translational (YdiV-SGFP, strain CR1417) fusions. Note that the YdiV-SGFP translational fusion is unable to repress FlhD_4_C_2_. Error bars indicate the standard deviations for three independent repeats. Download Figure S3, TIF file, 0.8 MB

Figure S4 The flagellar gene circuit does not exhibit hysteresis in Δ*ydiV* (CR1415) and Δ*fliZ* (CR1414) mutants. Class 2 P_*flhB*_ promoter activity as a function of anhydrotetracycline concentration (measure of FlhD_4_C_2_ expression inside the cells) in a P_*flhDC*_::*tetRA* Δ*ydiV* mutant initially off (A), P_*flhDC*_::*tetRA* Δ*ydiV* mutant initially on (B), P_*flhDC*_::*tetRA* Δ*fliZ* mutant initially off (C), and P_*flhDC*_::*tetRA* Δ*fliZ* mutant initially on (D) cells determined using flow cytometry. Download Figure S4, TIF file, 1.5 MB

Figure S5 Flagellar gene expression exhibits hysteresis even at higher concentrations of yeast extract. However, these experiments cannot be compared to each other, as the responses to yeast extract and aTc are not orthogonal: as yeast extract concentrations increase, expression from the aTc-inducible P_*tetA*_ promoter decreases for unknown reasons. (A to C) Class 2 P_*flhB*_ promoter activity as a function of anhydrotetracycline concentration (measure of FlhD_4_C_2_ concentration inside the cells) in a P_*flhDC*_::*tetRA* strain (strain CR1413), initially off (solid lines) or initially on (dashed line), grown with 0.5% yeast extract, 1% yeast extract, and 2% yeast extract. The data were normalized relative to those from the experiments using 0.2% yeast extract. (D) P_*tetA*_ promoter activity as a function of aTc and yeast extract measured using mCherry transcriptional fusion in a strain where the repressor TetR is produced independently from the P_*tetR*_ promoter (23). Data are averages from three independent repeats, and error bars indicate standard deviations. Download Figure S5, TIF file, 1.2 MB

Figure S6 Effect of parameter values on model simulations. The plots show the effect of varying one parameter at a time about its nominal value. The line darkness increases as the parameter values linearly increase. (Upper left) β_*F*_ from 0.1 to 1.5; (upper right) β_*Z*_ from 1 to 10; (lower left) γ_*C*_ from 60 to 400; (lower right) γ_*C*Y_ from 100 to 800. The nominal parameter values are as follows: β_*F*_ = 1, β_*Z*_ = 6, γ_*C*_ = 150, and γ_*C*Y_ = 500. Download Figure S6, TIF file, 3.2 MB

Figure S7 Introducing FliA does not alter behavior. (A) The plot shows the steady-state relative class 2 gene expression (*C*_2_) as a function of the YdiV expression rate (β_Y_) for the wild type (red line). In the absence of FliA or FliZ (lines overlap), no bistability is observed (gray line). (B) Model behavior as a function of the FlhD_4_C_2_ expression rate (β_*F*_) for the wild type (red line), the Δ*fliZ* Δ*fliA* mutant (gray line), and the Δ*ydiV* mutant (black line). The plot shows the steady-state relative class 2 gene expression (*C*_2_) as a function of the FlhD_4_C_2_ expression rate for a β_Y_ value of 5. The simulation involved the following dimensionless parameter values: β_*F*_ = 1, β_*Z*_ = 1, β_*A*_ = 0.5, β_*Z*_^*A*^ = 11, γ_*C*_ = 150, and γ_*C*Y_ = 500. Download Figure S7, TIF file, 1.4 MB
