# Supplementary material for: A Nutrient-Tunable Bistable Switch Controls Motility in Salmonella enterica Serovar Typhimurium
Source: mBio. 2014 Aug 26;5(5):e01611-14. doi: 10.1128/mBio.01611-14 (PMC4173784; doi:10.1128/mBio.01611-14)
Supplement: Text S1 — Detailed description of mathematical models discussed in the text. Download [file mbo004141956s1.pdf]

Supplementary Text: Description of mathematical models

1 Model ignoring contribution of FliA

The major assumptions used in formulating a model of bistability in the flagellar network are enumerated below.

1. Our analysis is limited to the steady-state behavior of the flagellar gene network. While the model was initially formulated as a set of coupled differential equations, our subsequent analysis considered only the steady-state behavior. We lacked sufficient information to parameterize the model for dynamic analysis. Specifically, many parameters were unknown or difficult to infer from the literature or our experiments.

2. The model is deterministic. Our goal in formulating the model was to understand the basic mechanism of bistability in flagellar gene expression. Specifically, we were interested in understanding which mechanisms generate the observed hysteresis. Moreover, as noted above, we lacked sufficient data to generate a comprehensive dynamic model, let alone a stochastic one.

3. The model does not include FlgM and the associated effects of secretion. Our justification was that FlgM does not affect bistability. Our data showed that FlgM was not required for bistability: a Δ*flgM* mutant behaved in a similar manner to the wild type (Figure 6A). Including FlgM in the model would only increase its complexity without any additional insights. Likewise, the model does not account for the FlgN, FlgK, and FlgL, known regulators of FlgM.

4. The model does not account for FliT. Similar to FlgM, our data showed that FliT was not required for bistability (Figure 6B).

5. The expression of the $FlhD_4C_2$ is known to be regulated by multiple factors [1]. We assumed that expression of the FlhD4C2 is constitutive and modeled it using a zero-order rate law. Our data provided no evidence that these regulatory factors are involved in bistability. Specifically, bistability was observed when we replaced the native P_{flhDC} promoter with an inducible one (Figure 5).

6. The model treats the $FlhD_4C_2$ heteromeric complex as a monomer. In particular, the model does not include a separate set of reactions describing the formation of the complex. We employed this assumption to limit the complexity of the model.

7. We assumed that YdiV binds to the $FlhD_4C_2$ complex with a 1:1 stoichiometry. In reality, two molecules of YdiV are known to bind to one $FlhD_4C_2$ molecule [2]. However, there is no evidence that binding is cooperative. In addition, we did not know how the binding of one

molecule of YdiV to the $FlhD_4C_2$ complex affects its activity relative to when two molecules were bound. Therefore, to limit the complexity of the model, we assumed a 1:1 stoichiometry.

8. YdiV promotes the degradation of the $FlhD_4C_2$ complex via ClpXP. We assumed that only the $FlhD_4C_2$ complex is targeted for proteolysis and not YdiV. This distinction, however, is effectively removed when we non-dimensionalized the model.
9. The model does not directly account for nutrients. Rather, their effect is modeled implicitly through the YdiV expression rate (the parameter β_Y).
10. The model does not distinguish between transcription and translation. The two processes were lumped together as a single reaction in the model, where the rate of protein synthesis is assumed implicitly to be proportional to the rate of mRNA synthesis. There is no evidence that bistability is regulated at the level of translation.
11. We employed simple Michaelis-Menten rate expressions to model the expression of FliZ and YdiV. We have no evidence to suggest more complex expression kinetics.
12. The model was initially formulated without including FliA. By ignoring FliA in this version of the model, we assumed that the FliZ is expressed from a class 2 promoter. As discussed in the main text, the model does not require FliA in order to generate bistability. However, our experimental results demonstrated that is in fact required (Figure 6). An extended version of the model account for FliA is discussed below.

The governing equations for the model are:

$$\frac{dF}{dt} = \beta_F - \gamma_F F - a_C FY + d_C C, \tag{1}$$

$$\frac{dY}{dt} = \frac{\beta_Y}{1 + K_Z Z} - \gamma_Y Y - a_C FY + d_C C + \gamma_C^{\text{ClpXP}} C, \tag{2}$$

$$\frac{dC}{dt} = a_C FY - d_C C - \gamma_C C - \gamma_C^{\text{ClpXP}} C, \tag{3}$$

$$\frac{dZ}{dt} = \frac{\beta_Z K_F F}{1 + K_F F} - \gamma_Z Z, \tag{4}$$

where t denotes time, F the concentration of the free $FlhD_4C_2$ complex (unbound to YdiV), Y the concentration of YdiV, C the concentration of the $FlhD_4C_2$-YdiV complex, and Z the concentration of FliZ. The parameter definitions are given in Table S1.

We simplified the model by limiting ourselves to the steady state. Under these conditions, the model reduces to the following set of algebraic equations:

$$0 = \beta_F - \gamma_F F - a_C FY + d_C C, \tag{5}$$

$$0 = \frac{\beta_Y}{1 + K_Z Z} - \gamma_Y Y - a_C FY + d_C C + \gamma_C^{\text{ClpXP}} C, \tag{6}$$

$$0 = a_C FY - d_C C - \gamma_C C - \gamma_C^{\text{ClpXP}} C, \tag{7}$$

$$0 = \frac{\beta_Z K_F F}{1 + K_F F} - \gamma_Z Z. \tag{8}$$

The variable C can be recast as a function of F and Y by solving equation (7):

$$C = \frac{a_C}{d_C + \gamma_C + \gamma_C^{\text{ClpXP}}} FY. \tag{9}$$

Parameter	Description
β_F	Expression rate for $FlhD_4C_2$
β_Y	Maximum expression rate for YdiV
β_Z	Maximum expression rate for FliZ
γ_F	$FlhD_4C_2$ degradation/dilution rate
γ_Y	YdiV degradation/dilution rate
γ_Z	FliZ degradation/dilution rate
γ_C	$FlhD_4C_2$-YdiV degradation/dilution rate (basal)
$\gamma_C^{\mathrm{ClpXP}}$	$FlhD_4C_2$-YdiV degradation rate (ClpXP)
a_C	$FlhD_4C_2$-YdiV association rate
d_C	$FlhD_4C_2$-YdiV disassociation rate
K_Z	Equilibrium constant for FliZ-*ydiV* promoter
K_F	Equilibrium constant for $FlhD_4C_2$-*fliAZ* class 2 promoter

Table S1: Parameter definitions

If the following definition is employed

$$K_C \triangleq \frac{a_C}{d_C + \gamma_C + \gamma_C^{\mathrm{ClpXP}}},$$

then equation (9) simplifies to $C = K_C F Y$. Note, if the parameters a_C and d_C are large relative to γ_C and $\gamma_C^{\mathrm{ClpXP}}$, then K_C is the equivalent to the equilibrium constant for the $FlhD_4C_2$-YdiV complex.

If equation (7) is added to equations (5) and (6), then the following simplified equations for F and Y are obtained:

$$\beta_F = \gamma_F F + \left(\gamma_C + \gamma_C^{\mathrm{ClpXP}}\right) K_C F Y, \tag{10}$$

$$\frac{\beta_Y}{1 + K_Z Z} = \gamma_Y Y + \gamma_C K_C F Y. \tag{11}$$

The model was further simplified by recasting in dimensionless form using the following parameter transformations and definitions:

$$F \leftarrow K_F F, \qquad Y \leftarrow K_C Y, \qquad Z \leftarrow K_Z Z,$$
$$\beta_F \leftarrow K_F \frac{\beta_F}{\gamma_F}, \qquad \beta_Z \leftarrow \frac{\beta_Z K_F}{\gamma_Z}, \qquad \beta_Y \leftarrow \frac{\beta_Y K_C}{\gamma_Y},$$
$$\gamma_C \leftarrow \frac{\gamma_C + \gamma_C^{\mathrm{ClpXP}}}{\gamma_F}, \qquad \gamma_{C_Y} \triangleq \frac{K_C \gamma_C}{K_F \gamma_Y}.$$

The dimensionless parameter γ_{C_Y} specifies the loss of YdiV due to association with $FlhD_4C_2$ – it is the analogue of γ_C for $FlhD_4C_2$. Note that values may be significantly different due to the rescaling of the variables. Specifically, these rates are given in terms of the respective dimensionless concentrations.

Application of these transformations and definitions results in the following set of equations

$$\beta_F = F + \gamma_C FY, \tag{12}$$

$$\frac{\beta_Y}{1+Z} = Y + \gamma_{C_Y} FY, \tag{13}$$

$$\frac{\beta_Z F}{1+F} = Z. \tag{14}$$

Equations (12)-(14) can be reduced to a cubic polynomial that potentially admits three real-valued solutions (equation not shown). Such a scenario would correspond to the coexistence of two stable steady states, with the third root representing the unstable steady state. In principle, we could determine the conditions for bistability by analytically solving the cubic equation or by analyzing of the associated Routh array. However, the polynomial is complex, such analysis was not informative (at least to the authors). In particular, the resulting expressions were unwieldy. As a consequence, we numerically solved equations (12)-(14) to determine whether the model is bistable.

The dimensionless model has five free parameter: β_F, β_Y, β_Z, γ_C, and γ_{C_Y}. Of these, β_Y is the natural bifurcation parameter as its value changes with nutrient concentrations: as nutrient increase, the value of β_Y decreases. The remaining parameters were chosen to generate bistability. The choice of these parameter values is not unique nor are they necessarily meant to reflect realistic values (in the dimensionless sense). Rather, our goal was to see if the model is sufficient to generate bistability. As we elaborated in the main text, the model is capable of generating bistability.

As the output of the model, we chose to plot the quantity

$$C_2 = \frac{F}{1+F},$$

as this provides a measure of class 2 gene expression, which was also the measured output in our experiments. This quantity is equivalent to normalized FliZ expression in this version of the model. This quantity is referred to relative class 2 gene expression in the Figure 5.

2 Model including contribution of FliA

We can extend the model to include FliA (σ^{28}) using the following equations:

$$\frac{dF}{dt} = \beta_F - \gamma_F F - a_C FY + d_C C, \tag{15}$$

$$\frac{dY}{dt} = \frac{\beta_Y}{1+K_Z Z} - \gamma_Y Y - a_C FY + d_C C + \gamma_C^{\text{ClpXP}} C, \tag{16}$$

$$\frac{dC}{dt} = a_C FY - d_C C - \gamma_C C - \gamma_C^{\text{ClpXP}} C, \tag{17}$$

$$\frac{dZ}{dt} = \frac{\beta_Z K_F F}{1+K_F F} + \frac{\beta_Z^A K_A A}{1+K_A A} - \gamma_Z Z, \tag{18}$$

$$\frac{dA}{dt} = \frac{\beta_A K_F F}{1+K_F F} - \gamma_A A. \tag{19}$$

where A denotes the concentration of FliA. The new parameters are listed in Table S2. In formulating this model, we have made the following additional assumptions:

Parameter	Description
β_Z	Maximum expression rate for FliZ due to class 2 *fliAZ* promoter
β_Z^A	Basal expression rate for FliZ due to class 3 *fliAZ* promoter
β_A	Maximum expression rate for FliA
γ_A	FliA degradation/dilution rate
K_A	Equilibrium constant for FliA-*fliAZ* class 3 promoter

Table S2: Parameter definitions for model extension

1. FliA is not translated from class 3 mRNA transcripts [3]. Its expression is solely dependent on $FlhD_4C_2$.

2. The class 2 and class 3 P_{fliAZ} promoters are independent. The expression rate is modeled as the sum of two Michaelis-Menten rate laws. Employing a multisite rate law did not yield any significant differences.

The steady-state version of the extended model described by equations (15)-(19) can be simplified as before with the additional dimensionless parameter transformations:

$$A \leftarrow K_A A, \qquad \beta_Z^A \leftarrow \frac{\beta_Z^A K_Z}{\gamma_Z}, \qquad \beta_A \leftarrow \frac{\beta_A K_A}{\gamma_A}.$$

Applying these transformation yields the following equations:

$$\beta_F = F + \gamma_C F Y, \tag{20}$$

$$\frac{\beta_Y}{1+Z} = Y + \gamma_{C_Y} F Y, \tag{21}$$

$$\frac{\beta_Z F}{1+F} + \frac{\beta_Z^A A}{1+A} = Z, \tag{22}$$

$$\frac{\beta_A F}{1+F} = A. \tag{23}$$

We can eliminate the variable by substituting equation (23) into equation (22), yielding the equation:

$$\frac{\beta_F F}{1+F} + \frac{\beta_Z^A \beta_A F}{1+(1+\beta_A)F} = Z. \tag{24}$$

By simple inspection, we can see that FliA (A) has the effect of amplifying the expression of FliZ (Z) with respect to $FlhD_4C_2$ (F). As a consequence, the value for β_Z need not be so large as the other parameters – β_Z^A and β_A – also additively contribute to FliZ expression. As discussed the main text, FliAs contribution is indirect through enhanced expression of FliZ.

References

[1] O. A. Soutourina and P. N. Bertin. Regulation cascade of flagellar expression in Gram-negative bacteria. *FEMS Microbiol. Rev.*, 27(4):505–523, Oct 2003.

[2] A. Takaya, M. Erhardt, K. Karata, K. Winterberg, T. Yamamoto, and K. T. Hughes. YdiV: a dual function protein that targets FlhDC for ClpXP-dependent degradation by promoting release of DNA-bound FlhDC complex. *Mol. Microbiol.*, 83(6):1268–1284, Mar 2012.

[3] Y. Tanabe, T. Wada, K. Ono, T. Abo, and K. Kutsukake. The transcript from the (28)-dependent promoter is translationally inert in the expression of the (28)-encoding gene fliA in the fliAZ operon of Salmonella enterica serovar Typhimurium. *J. Bacteriol.*, 193(22):6132–6141, Nov 2011.
