# Supplementary figures and images for: A Nutrient-Tunable Bistable Switch Controls Motility in Salmonella enterica Serovar Typhimurium
Source: mBio. 2014 Aug 26;5(5):e01611-14. doi: 10.1128/mBio.01611-14 (PMC4173784; doi:10.1128/mBio.01611-14)

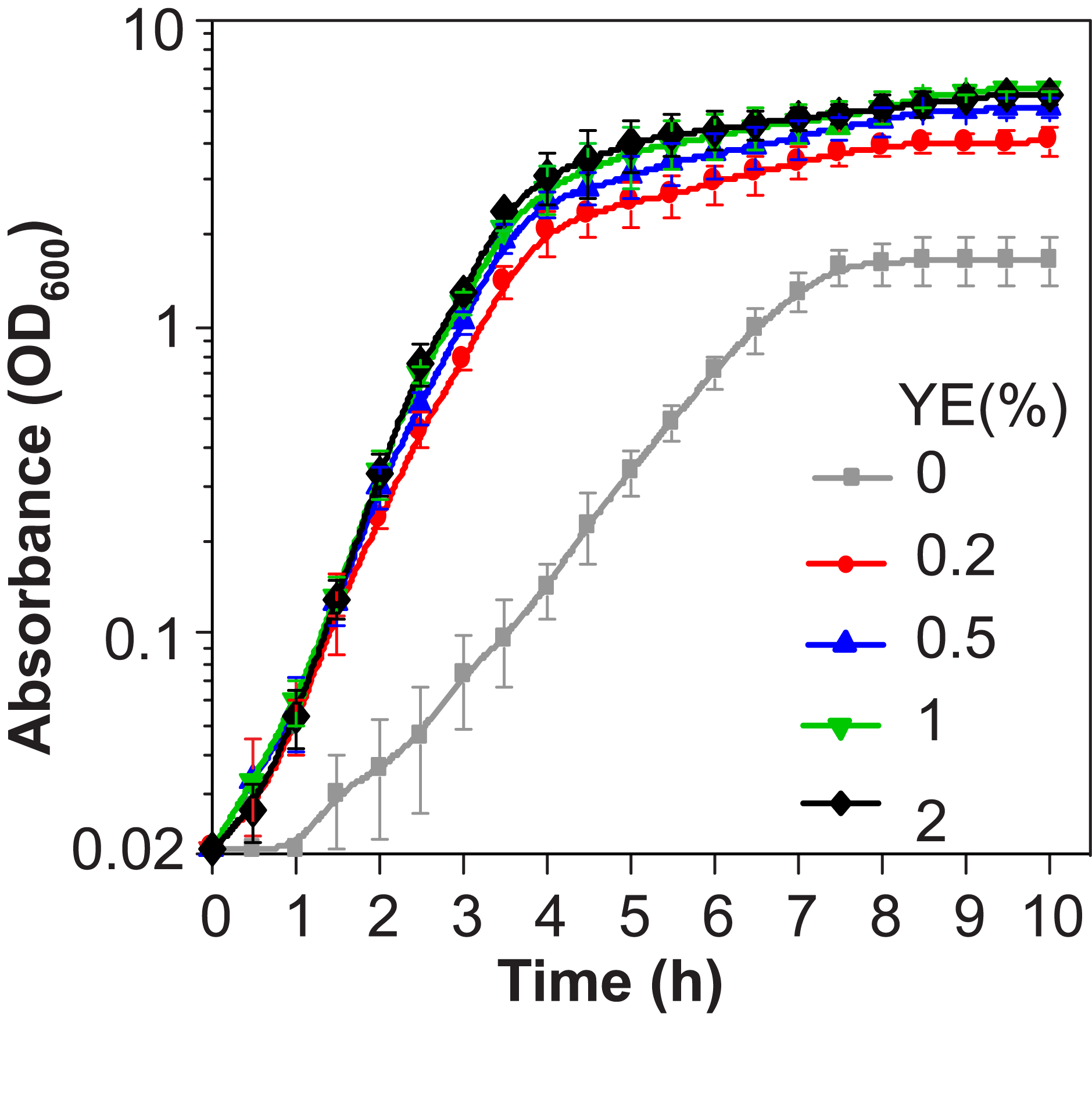

Supplement: Figure S1 — Growth curves of wild-type cells (strain 14028) at different concentrations of yeast extract. Error bars indicate standard deviations for three independent repeats. Download [file mbo004141956sf01.tif]

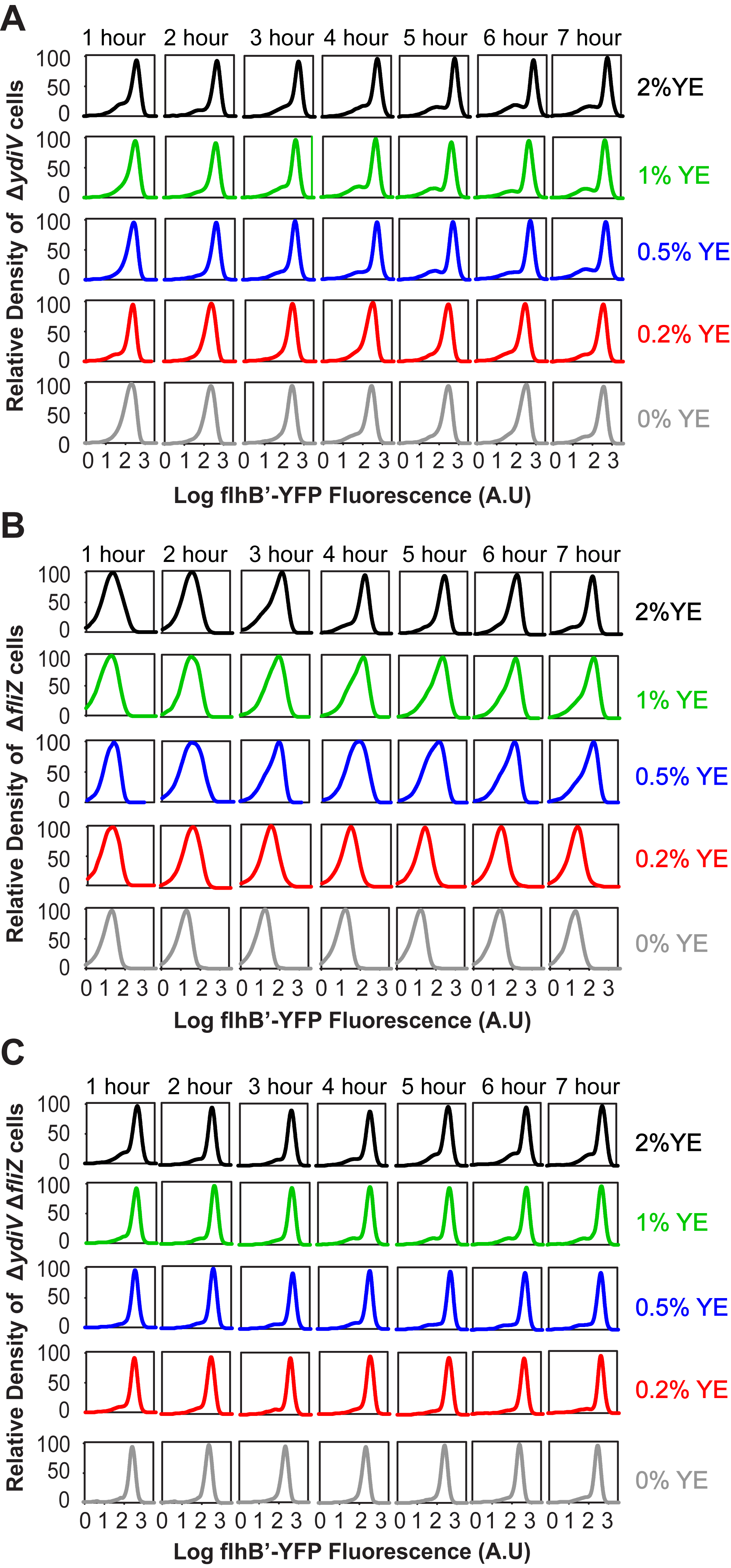

Supplement: Figure S2 — The class 2 gene expression profile is unimodal in the absence of two antagonizing proteins, YdiV and FliZ. (A to C) Class 2 PflhB promoter activity as a function of time and yeast extract concentration, as determined by flow cytometry in ΔydiV, ΔfliZ, and ΔydiV ΔfliZ mutants (strains CR1407, CR1408, and CR1409, respectively). Download [file mbo004141956sf02.tif]

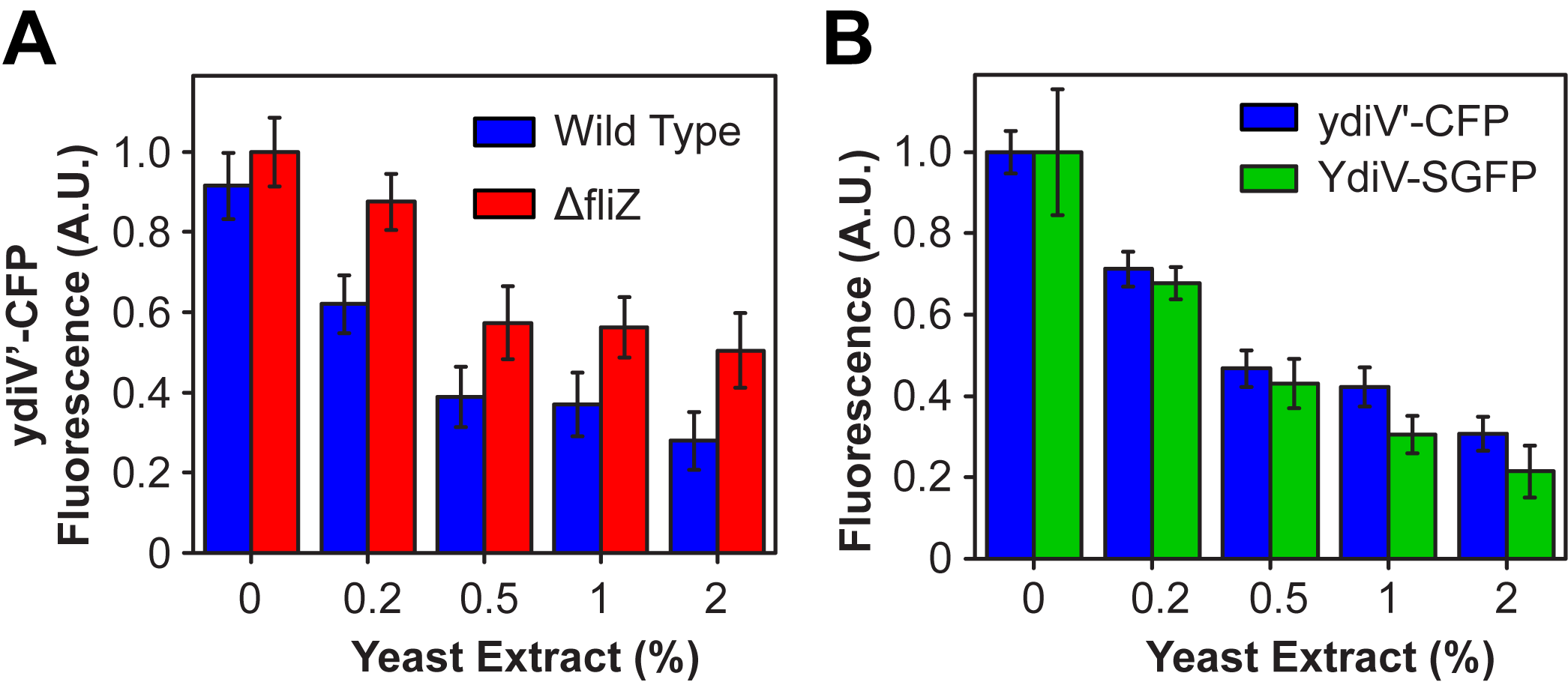

Supplement: Figure S3 — (A) YdiV transcription is enhanced under nutrient-limited conditions, and its transcription is repressed by FliZ. PydiV promoter activity as a function of yeast extract in wild-type and ΔfliZ strains (PydiV-CFP plasmid in strains 14028 and CR201, respectively). (B) YdiV is regulated at the transcriptional level. Comparison of YdiV transcriptional (ydiV′-CFP) and translational (YdiV-SGFP, strain CR1417) fusions. Note that the YdiV-SGFP translational fusion is unable to repress FlhD4C2. Error bars indicate the standard deviations for three independent repeats. Download [file mbo004141956sf03.tif]

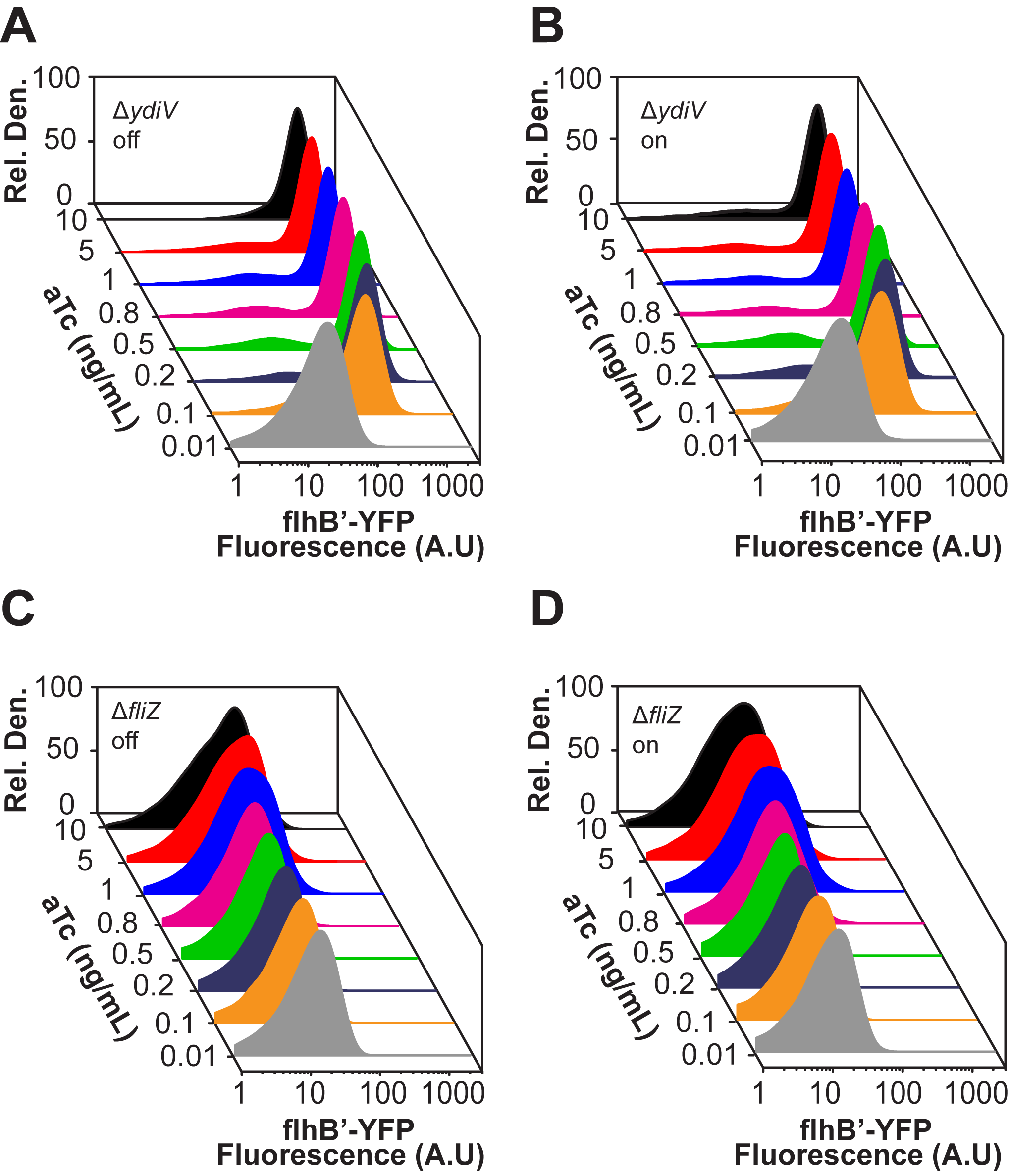

Supplement: Figure S4 — The flagellar gene circuit does not exhibit hysteresis in ΔydiV (CR1415) and ΔfliZ (CR1414) mutants. Class 2 PflhB promoter activity as a function of anhydrotetracycline concentration (measure of FlhD4C2 expression inside the cells) in a PflhDC::tetRA ΔydiV mutant initially off (A), PflhDC::tetRA ΔydiV mutant initially on (B), PflhDC::tetRA ΔfliZ mutant initially off (C), and PflhDC::tetRA ΔfliZ mutant initially on (D) cells determined using flow cytometry. Download [file mbo004141956sf04.tif]

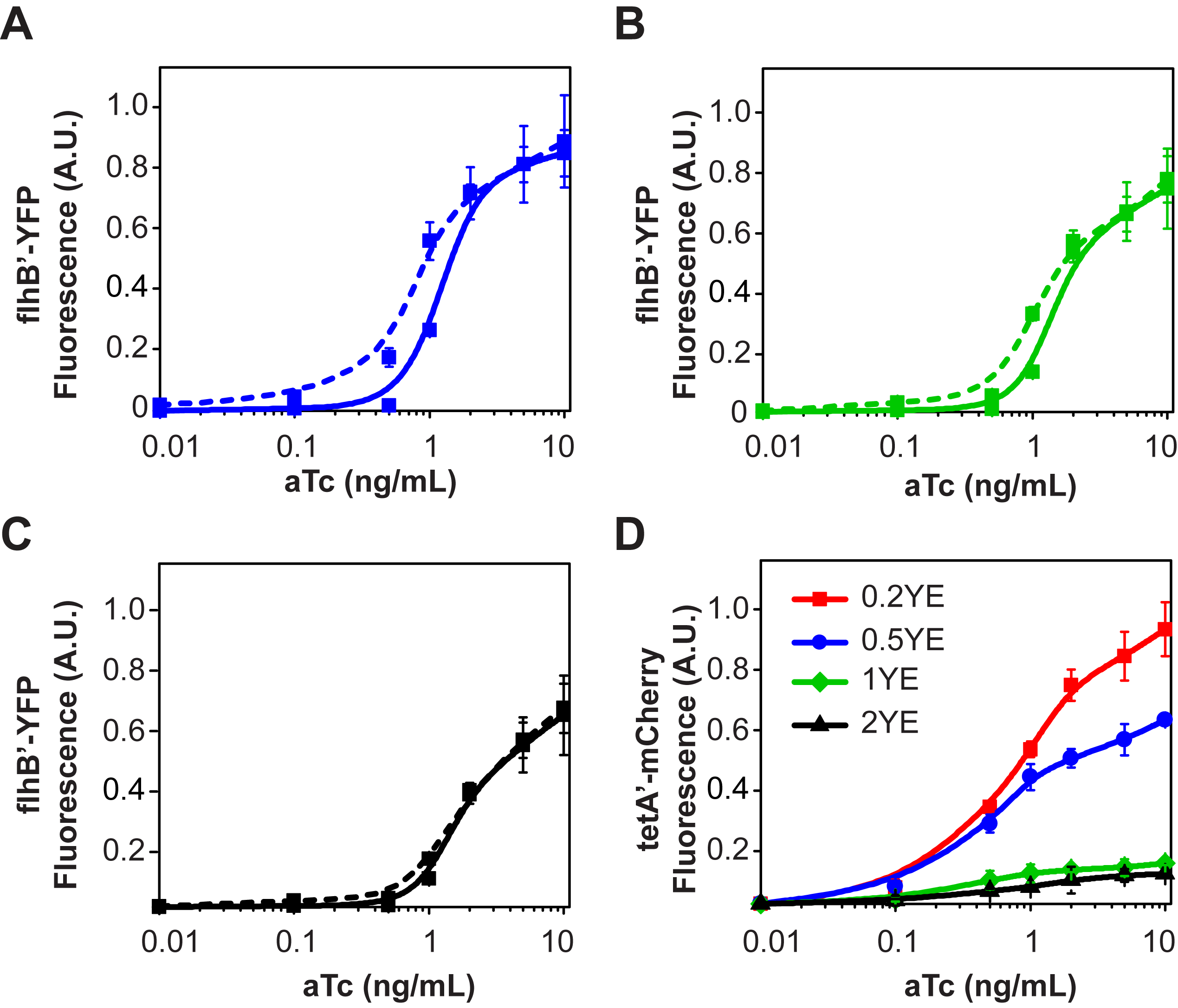

Supplement: Figure S5 — Flagellar gene expression exhibits hysteresis even at higher concentrations of yeast extract. However, these experiments cannot be compared to each other, as the responses to yeast extract and aTc are not orthogonal: as yeast extract concentrations increase, expression from the aTc-inducible PtetA promoter decreases for unknown reasons. (A to C) Class 2 PflhB promoter activity as a function of anhydrotetracycline concentration (measure of FlhD4C2 concentration inside the cells) in a PflhDC::tetRA strain (strain CR1413), initially off (solid lines) or initially on (dashed line), grown with 0.5% yeast extract, 1% yeast extract, and 2% yeast extract. The data were normalized relative to those from the experiments using 0.2% yeast extract. (D) PtetA promoter activity as a function of aTc and yeast extract measured using mCherry transcriptional fusion in a strain where the repressor TetR is produced independently from the PtetR promoter (23). Data are averages from three independent repeats, and error bars indicate standard deviations. Download [file mbo004141956sf05.tif]

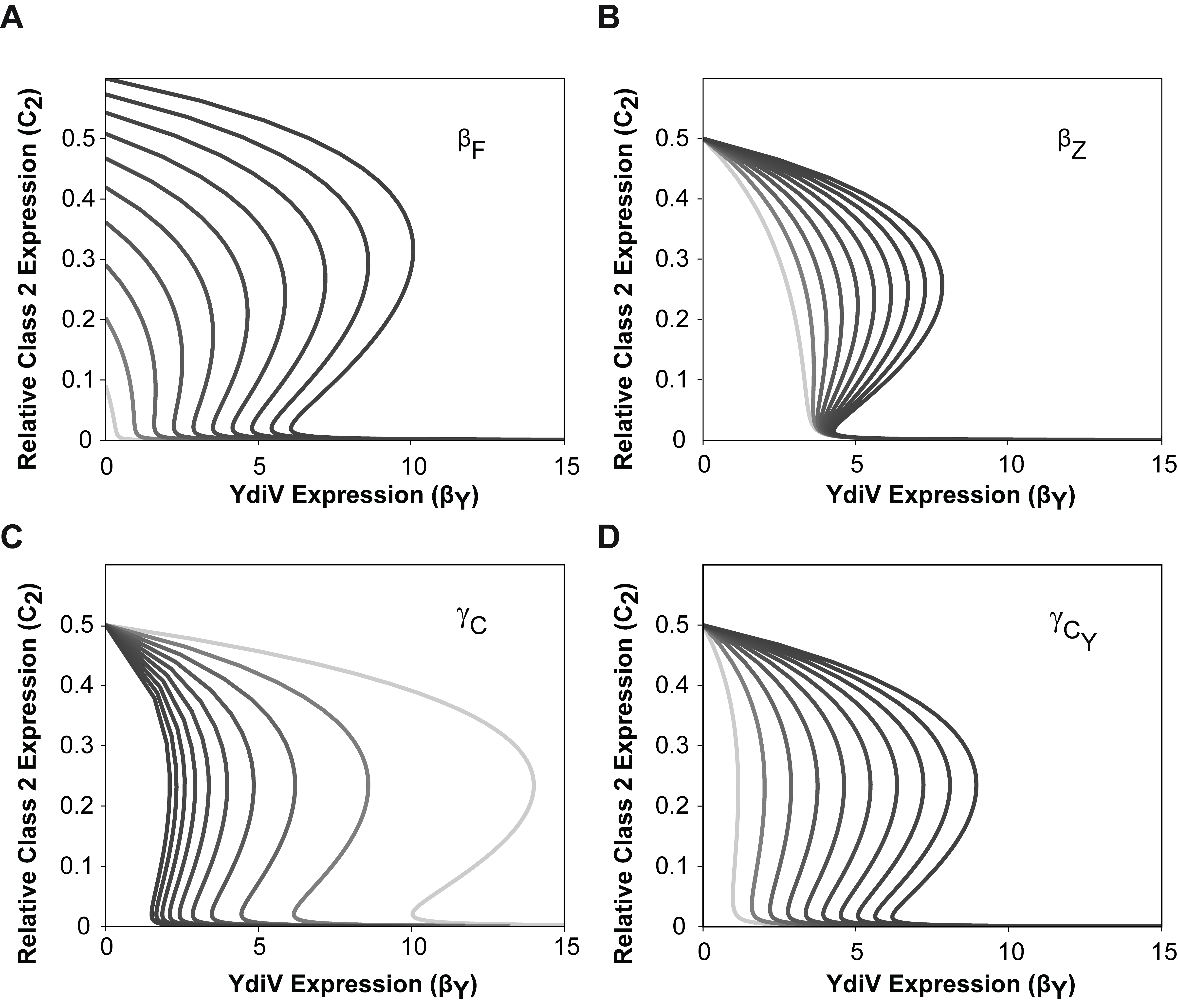

Supplement: Figure S6 — Effect of parameter values on model simulations. The plots show the effect of varying one parameter at a time about its nominal value. The line darkness increases as the parameter values linearly increase. (Upper left) βF from 0.1 to 1.5; (upper right) βZ from 1 to 10; (lower left) γC from 60 to 400; (lower right) γCY from 100 to 800. The nominal parameter values are as follows: βF = 1, βZ = 6, γC = 150, and γCY = 500. Download [file mbo004141956sf06.tif]

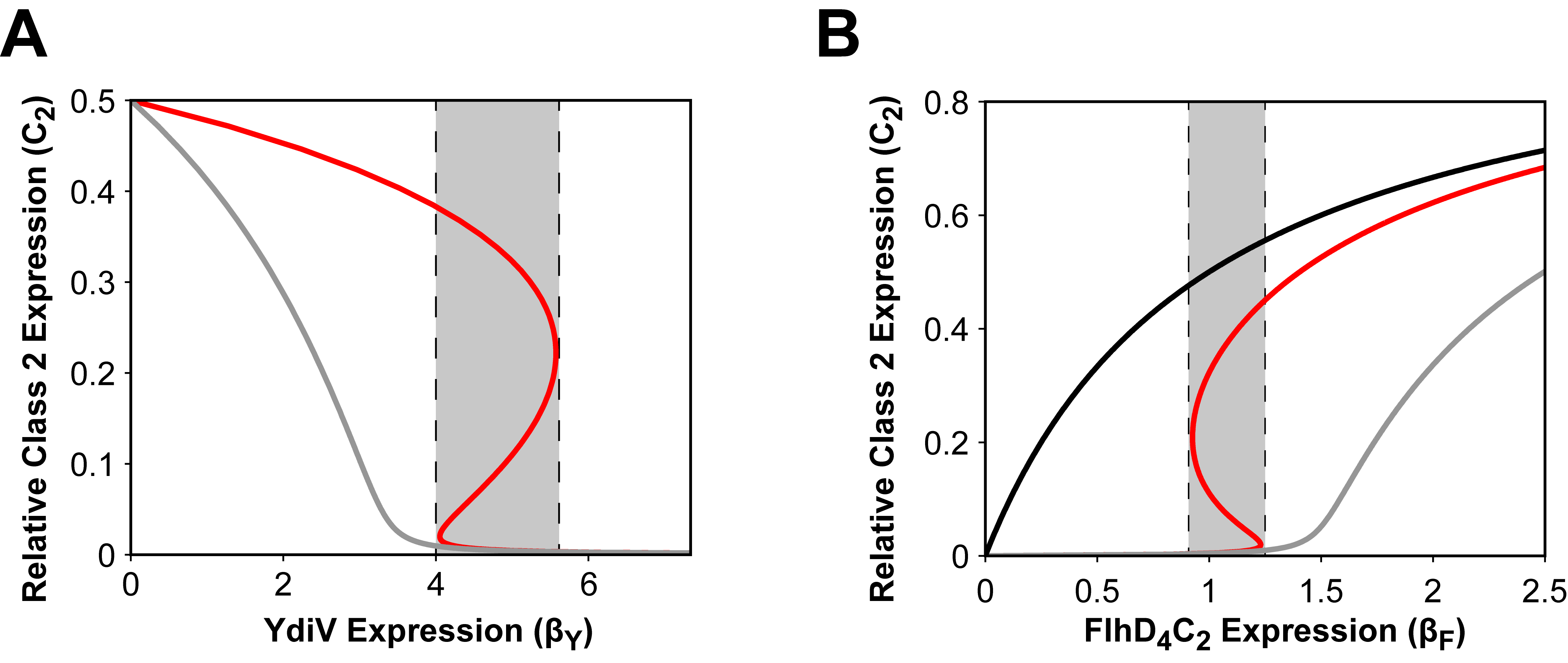

Supplement: Figure S7 — Introducing FliA does not alter behavior. (A) The plot shows the steady-state relative class 2 gene expression (C2) as a function of the YdiV expression rate (βY) for the wild type (red line). In the absence of FliA or FliZ (lines overlap), no bistability is observed (gray line). (B) Model behavior as a function of the FlhD4C2 expression rate (βF) for the wild type (red line), the ΔfliZ ΔfliA mutant (gray line), and the ΔydiV mutant (black line). The plot shows the steady-state relative class 2 gene expression (C2) as a function of the FlhD4C2 expression rate for a βY value of 5. The simulation involved the following dimensionless parameter values: βF = 1, βZ = 1, βA = 0.5, βZA = 11, γC = 150, and γCY = 500. Download [file mbo004141956sf07.tif]
